# Photonic Molecularly Imprinted Polymer Film for the Detection of Testosterone in Aqueous Samples

**DOI:** 10.3390/polym10040349

**Published:** 2018-03-22

**Authors:** Abbas J. Kadhem, Shuting Xiang, Susan Nagel, Chung-Ho Lin, Maria Fidalgo de Cortalezzi

**Affiliations:** 1 Department of Civil and Environmental Engineering, University of Missouri, Columbia, MO 65211, USA; ajkqmb@mail.missouri.edu (A.J.K.); sx6cf@mail.missouri.edu (S.X.); 2 Department of Obstetrics, Gynecology and Women’s Health, University of Missouri, Columbia, MO 65211, USA; nagels@health.missouri.edu; 3 School of Natural Resources, University of Missouri, Columbia, MO 65211, USA; linchu@missouri.edu

**Keywords:** molecularly imprinted polymer, testosterone, colloidal crystal, photonic films, Bragg diffraction

## Abstract

The detection of testosterone in aqueous solutions is a difficult task due to the low concentration levels that are relevant in environmental and physiological samples. Current analytical methods are expensive and/or complex. To address this issue, we fabricated a molecularly imprinted polymer (MIP) photonic film for the detection of testosterone in water. The films were obtained using colloidal crystals as templates for the pore morphology. Monodispersed silica particles with an average diameter 330 nm were used to obtain the colloidal crystal by vertical deposition. A solution of acrylic acid with testosterone as the imprinted template was infiltrated in the colloidal crystal and polymerized via bulk polymerization; the particles were then removed by acid etching and the testosterone eluted by a suitable solvent. The material was characterized by FTIR, swelling experiments and microscopy; MIPs were investigated by equilibrium rebinding, kinetics and reuse experiments. The results showed that the MIPs exhibited selectivity to the template, a 30-min equilibration time and stability after at least six cycles of use and regeneration. After incubation, the reflectance spectra of the films showed a shift of the Bragg diffraction peak that correlated with testosterone concentration in the 5–100 ppb range.

## 1. Introduction

Testosterone is the primary androgen hormone that has a significant effect on human health [1]. The average level of testosterone in a healthy adult male is 300–1000 ng/dL [2]. It has been used illegally by athletes as a result of its anabolic effect, which is related to muscle mass and strength growth [3]. The low concentration of testosterone makes it challenging to find suitable, efficient, and economical methods of detection and quantification. Nowadays, many analytical methods are used to monitor testosterone in urine and blood samples such as Gas Chromatography-Mass Spectrometry (GC-MS) [4,5], Isotope-Dilution Gas Chromatography-Mass Spectrometry (ID/GC-MS) [6], and Liquid Chromatography-Mass Spectrometry (LC-MS-MS) [7]. These methods are complicated, expensive, time consuming, and operator training is required [8], which leads to the need for a simple, rapid, and low cost method to detect the testosterone in aqueous samples.

Molecularly imprinted polymers (MIPs) constitute a feasible alternative. Molecular Imprinting (MI) is a technology that allows the fabrication of a polymer material with the ability to selectively adsorb the target molecules as a result of the highly specific sites formed during fabrication [9]. The key steps to the process of molecularly imprinted polymers include: integration of the target into the monomer solution, polymerization to carefully design the spatial distribution of template molecules inside the polymeric matrix, template removal to provide cavities having the same morphology of the target, and rebinding the target in a mechanism similar to the “key-lock” principle of enzymes [10]. Synthetic MIPs have shown advantages over their biological counterparts including improved stability, simple preparation, excellent ability to capture small molecules, reusability and longer shelf-lives [11]. MIPs have been investigated extensively for their use in analytical chemistry and sensing in environmental, chemical and biomedical fields, but more research is needed to address the significant challenges still preventing large scale application of the technology and reaching its full capabilities [12].

Several methods were used to analyze the testosterone using MIPs, mostly as an adsorption or concentrating media in combination with an analytical instrument for quantification, including microring resonator [1], High Performance Liquid Chromatography-Mass Spectrometry (HPLC-MS-MS) [13], High Performance Liquid Chromatography-Solid Phase Extraction (HPLC-SPE) [14], (SPME-GC-MS) [15], and Liquid Chromatography-Mass Spectrometry (LC-MS-MS) [16]. However, these methods are still complicated, expensive and time consuming. An optical sensor based in MIPs is a promising method, since it does not need a second analytical step. The methodology relies on the adsorption of the target molecule, i.e., testosterone, to an optical active thin film and the change in the light reflectance properties that is produced after adsorption to detect the binding of the target [17]. Photonic MIPs have been reported for the detection of contaminants in water and food, including bisphenol A [18], cinchonice [19], tetracycline [20], vanillin [21] and cholesterol [22] and others.

In this work, a photonic sensor based on a combination between colloidal crystal from silica nanoparticles, molecularly imprinted polymers, and inverse opal technique was fabricated. First, a thin MIP porous film was obtained with a morphology corresponding to the inverse of a silica particles colloidal crystal. Secondly, an optical sensor (supported MIP film) was developed and tested. This optical sensor had the ability to swell or shrink in aqueous solution upon molecular recognition or environmental conditions leading to a change in the optical properties. Unsupported MIP characterization and validation experiments were conducted aided by HPLC determination of testosterone in the test samples, prior to the use of the supported MIP film to detect the template by reflectance. The technique was used for the first time to create a testosterone optical sensor, with detection and quantification capabilities in aqueous samples.

## 2. Materials and Methods

Tetraethoxysilane (TEOS), ammonia solution (25% in water), ethanol (99.5%, 200 proof), acrylic acid (AA) (99%), methacrylic acid (MAA) (99%), ethylene glycol dimethacrylate (EGDMA) (98%), 2,2′-azobisisobutyronitrile (AIBN) (98%), testosterone (99%), bisphenol A (99%), flutamide (100%) and 17 beta-estradiol (98%) were supplied by Sigma-Aldrich, St. Louis, MO, USA. Hydrofluoric acid (HF) (49%), acetic acid (96%), acetonitrile and phosphate buffer solution were purchased from Fisher, Fair Lawn, NJ, USA. Ultra-pure water (18.2 MΩ·cm at 25 °C) was obtained from a Thermo Scientific™ Barnstead™ E-Pure™ Ultrapure Water Purification System (Waltham, MA, USA). All chemicals were purchased as reagent grade and used without further purification.

**Figure 1:**
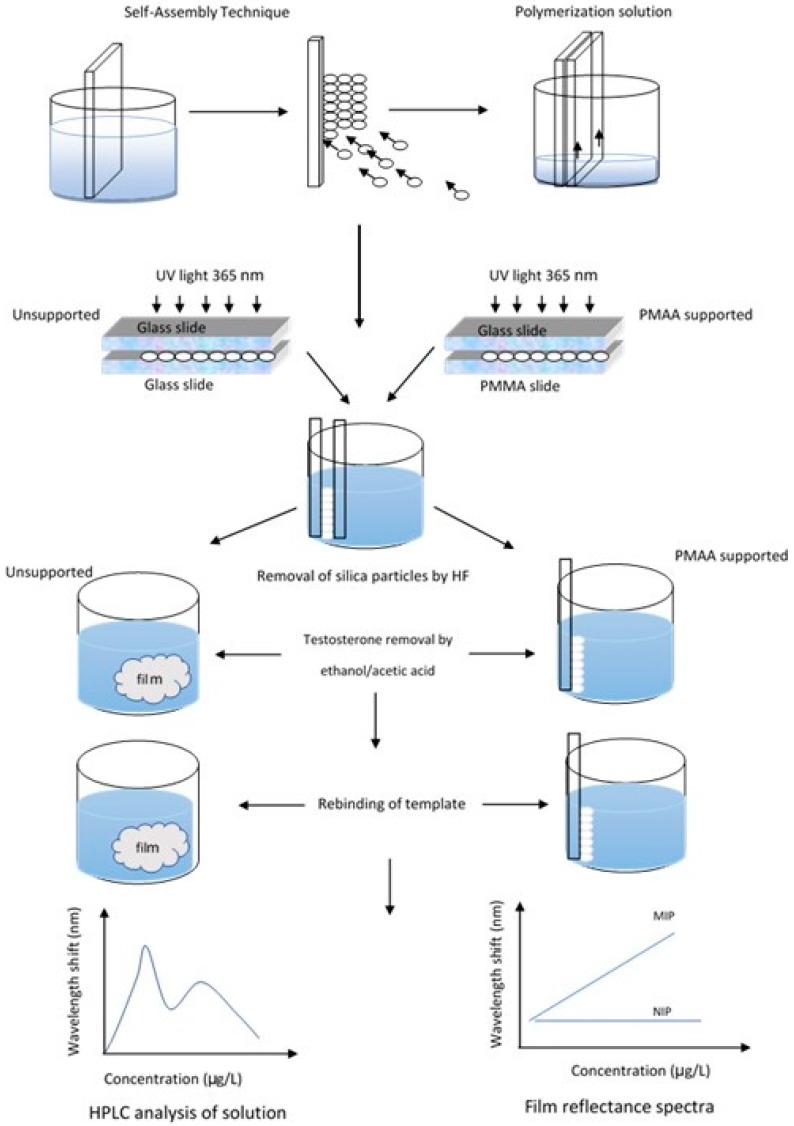
Schematic representation of the fabrication process and research design.

The research approach is schematically shown in Figure 1. A colloidal crystal, fabricated by vertical self-assembly, was secured between two slides, infiltrated by the polymerization solution, and polymerized under UV-light. Immersion in HF resulted in detachment of the glass and removal of the silica particles. When two glass slides were used, unsupported films were obtained, while poly (methyl methacrylate)-supported MIPs were fabricated when the second slide was plastic. The unsupported material was used for the characterization and binding experiments, while the concentration of testosterone in solution was measured by HPLC; the photonic properties were investigated in the supported films, recording their UV-visible reflectance spectra.

### 2.1. Synthesis of Porous MIPs

Silica particles were synthesized by a modified Stöber method [23]. Briefly, ethanol (200 mL) was mixed in a flask and stirred with ammonia (22 mL) at 300 rpm for approximately 10 min. Then, TEOS (14 mL) was added rapidly and the mixture allowed to react for 12 h. Particles were centrifuged and re-dispersed in ethanol once and in distilled water twice to remove residual reactants. For the fabrication of colloidal crystals, a clean microscope glass slide (Fisher Scientific, Pittsburgh, PA, USA) with dimensions 1 × 13 × 76 mm was placed vertically in a vial containing a suspension of silica particles (volume fraction 0.1%) in ethanol; after the volatilization of ethanol, colloidal crystals were self-assembled on both sides of the glass slides.

The MIP films were fabricated using a non-covalent self-assembly approach, filling the void volume in the colloidal crystal with a polymerization solution. The solution was prepared with 5 mg of testosterone dissolved in 2 mL of acetonitrile; the functional monomer AA or MAA (400 µL) was added and the liquid stirred in the dark for 3 h. A volume of 400 µL of each monomer was added to the polymerization mixture (0.006 mM of AA, 0.005 mM of MAA). Then, 500 µL of the crosslinker EGDMA and 9 mg of AIBN as initiator, were added.

A second clean glass slide was placed on top of the one containing the colloidal crystal and the two slides were firmly held together forming a “sandwich”-type structure. One end of this structure was put in contact with the polymerization solution and the liquid was allowed to rise by capillary forces until it completely filled the void volume within the colloidal crystal. The polymerization was completed under UV light at λ = 365 nm, Cole Parmer lamp (Vernon Hills, IL, USA), I = 2 mW·cm^−2^, for 8 h at room temperature. Non-imprinted polymers (NIPs) were prepared as a control following the same procedure as MIPs with the exception of testosterone addition.

The silica particles, now entrapped within the polymer, were etched away by immersion in 5% HF for 12 h, followed by exhaustive rinsing with deionized water. This step also produced the detachment of the film from the glass slide. Finally, the resulting films were washed with 1:1 (*v*:*v*) acetic acid: ethanol solutions for 30 min to remove the testosterone. The washing procedure was repeated 6 times, after which the films were subjected to a final rinse with ethanol to remove any remaining acid. Supported thin films were fabricated following a similar methodology as described above for the self-standing polymers, but, in this approach, poly(methyl methacrylate) (PMMA) slides of the same size (ePlastics, San Diego, CA, USA) were used as the second slide to make the “sandwich” structure; additionally, the elution of the testosterone was conducted using 1:9 (*v*:*v*) acetic acid: ethanol, changing the solvent every 30 min for 5 h, since the solution used for the unsupported MIPs damaged the PMMA slides. The inverse opal film was firmly attached to the surface of PMMA support slides.

### 2.2. Characterization

The size and morphology of silica particles and the porous films were investigated by electron microscopy in a FEI Quanta 600 FEG (ThermoFisher Scientific, Hillsboro, OR, USA) Environmental Scanning Electron Microscopy (ESEM). The specimens were mounted on stubs using carbon tape and covered with a thin conductive layer of gold (1.5–3.0 nm) by an Emitech K575x sputter coater (Quorum Technologies Ltd., Ashford, Kent, UK). ImageJ software version 1.50 (National Institutes of Health, NIH) was used to analyze the SEM images. In the determination of particle size, at least 300 particles were measured from 3 different images. The size of silica particles was confirmed by Dynamic Light Scattering (DLS) on a Zetasizer Nano ZS instrument (Malvern Instruments, Malvern, UK).

Equilibrium swelling experiments were conducted on NIPs, synthesized from AA and MAA, in phosphate buffer at two pHs (6 and 8), at 25 °C. The polymers were swollen in solutions for 72 h at room temperature to reach equilibrium, and the degree of swelling was determined gravimetrically. The swelling ratio *(SR*) was calculated from the following expression:

(1) $$SR=\left(m_{s}-m_{d}\right)/m_{d}$$

where *m_s_* is the mass of the swollen film at equilibrium and *m_d_* the mass of the lyophilized films.

Fourier Transformed Infrared Spectroscopy (FTIR) spectra were collected in a Cary 660 spectrometer (Agilent Technologies, Santa Clara, CA, USA) between the wavelengths of 4000–400 cm^−1^.

The recognition binding experiments were conducted by immersing 5 mg of lyophilized MIP or NIP film in testosterone solutions of variable concentration, from 0.1 ppm to 20 ppm, for 24 h when equilibrium was assumed. The residual concentration of the solution was determined by HPLC, and the recognition capacity was calculated from Equation (2):

(2) RC=(Ci−Ce)Vt/m

where *Ci*, *Ce* are the initial and the final concentrations of testosterone (in mg·mL^−1^), respectively; *Vt* is the volume of solution (in mL), and *m* is the mass of the film (in g). The imprinting polymer efficiency (*IE*) was calculated as:

(3) $$IE=\frac{RC_{MIP}}{RC_{NIP}}$$

where *RC_MIP_* and *RC_NIP_* are the recognition capacities of *MIP* and *NIP*, respectively.

The kinetics of testosterone binding was investigated for unsupported MIPs in 5 ppm testosterone solutions. During incubation, samples were taken periodically and residual testosterone concentration was measured by HPLC. Additionally, the kinetics of the optical response (shift of the Bragg’s diffraction peak) of supported films in a 1 ppm testosterone solution was assessed by collecting reflectance spectra for the MIP film periodically during incubation.

The desorption of testosterone after incubation was analyzed using unsupported films. Once equilibrium was reached (i.e., 24 h incubating time), the films were immersed in pure water for another 24 h and then the liquid analyzed for testosterone. The experiment was conducted with initial testosterone concentrations between 0.1 ppm and 1 ppm.

### 2.3. Analytical Method

The testosterone solutions were analyzed by High-Performance Liquid Chromatography (HPLC) in a Shimadzu LC-2010A HT (Kyoto, Japan). A Kinetex^®^ C18 stainless steel column (Phenomenex, Torrance, CA, USA) with 2.6 µm particles size, 100 mm length × 4.6 mm internal diameter was used, with a flow rate of 0.5 mL/min. The system was washed first with 50% acetonitrile and 50% of 1% phosphoric acid, and the column was washed with 2% acetonitrile and 98% of 1% phosphoric acid solution. The injection volume was 20 µL; detection was performed by UV-Vis absorption at a wavelength of 265 nm; the technique has a detection limit of 10 ppb.

### 2.4. Reflectance Analysis

Testosterone adsorption into the binding sites resulted in a change in Bragg diffraction of the polymer due to swelling or shrinking of the hydrogel film and refractive index changes, and therefore a clear optical signal can be detected. UV-Vis reflectance spectra of the films were recorded and their respective *λ_max_* was correlated to solution concentration. The diffraction peak *λ_max_* for the porous hydrogel is given by the Bragg equation:

(4) $$\lambda_{max}=1.633\left(\frac{d}{m}\right)\left(\frac{D}{D_{0}}\right){\left(n_{a}^{2}-\sin\theta^{2}\right)}^{0.5}$$

where *d* is the sphere diameter of the colloidal silica particle, *m* is the order of Bragg diffraction, (*D/D*_0_) is the degree of swelling of the gel (*D* and *D*_0_ denote the diameters of the gel in the equilibrium state at a certain condition and in the reference state, respectively), *n_a_* is the average refractive index of the porous gel at a particular condition, and *θ* is the angle of incidence.

The reflectance of the films was measured over a wavelength range of 200–800 nm, using a double-beam UV–visible spectrophotometer (Cary 60, Varian, Palo Alto, CA, USA) with a Harrick Scientific’s Specular Reflection Accessory (ERA-30G) at a fixed angle of 30°.

## 3. Results

### 3.1. Characterization of Silica Particles and Films

**Figure 2:**
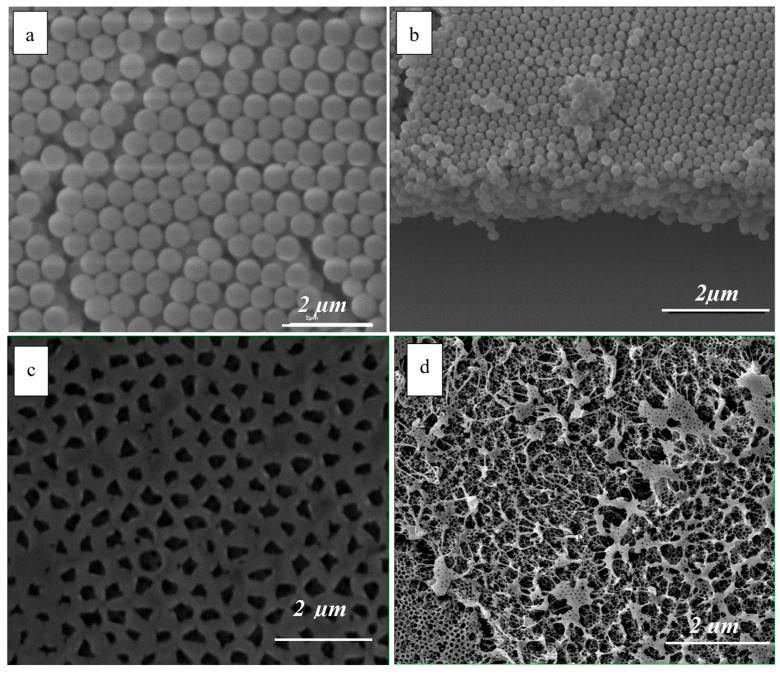
SEM images of: (**a**) colloidal crystal top layer; (**b**) colloidal crystal showing cross section; (**c**) porous structure (top view) of the MIP film; (**d**) internal morphology of the MIP film.

The monodispersed silica particles were prepared with careful control of the reaction conditions in order to achieve the desired narrow size distribution. Figure 2a,b shows representative SEM images of the top view and cross section of the obtained colloidal crystals. Size measurements from the SEM images (top views only) showed a narrow size distribution of the particles, with an average radius of 330 nm and standard deviation of 8 nm. Only particles with a standard deviation below 5% were used in the deposition experiments.

The average particle size was confirmed by DLS measurements that gave an average hydrodynamic diameter of 375 ± 7 nm and a polydispersivity index of 0.012. The two measurements are in reasonably good agreement considering experimental error and the fact that DLS commonly overestimates size due to the effect of the solvent in the hydrodynamic diameter [24].

Colloidal crystals were prepared by vertical self-assembly from 0.1% volume fraction suspensions; the particle concentration was chosen to achieve a desired film thickness. Jiang et al. [25] established that the relationship between initial volume fraction of the nanoparticle suspension and the thickness of the self-assembled crystal can be described by Equation (5):

(5) $$K=\frac{\beta L\emptyset }{0.605\;d\left(1-\emptyset \right)}$$

where *K* is the number of layers, *L* is the meniscus height, *β* is the ratio between the velocity of a particle in solution and the fluid velocity and is taken to be 1, *d* is the particles diameter, and ∅ is the particle volume fraction in solution. The calculated number of layers according to Equation (5), with *L* = 3500 µm, ∅ = 0.001 and *d* = 330 nm, was 17, while the number of layers observed from SEM images was between 13–15 layers. A thicker film would offer a larger total surface area, and, therefore, more binding sites, but diffusion into the lower cavities would slow down equilibration of the film, and, consequently, the response. Furthermore, as colloidal crystal grew thicker, they became unstable and partially detached from the slides, leading to faulty films. Thus, the thinner film that produces a measurable response within the range of values of interest would represent the optimal condition. The thickness of the MIPs was estimated to be approximately 5 µm, given the average number of silica particle layers observed in the colloidal crystals and the measurements of the silica particle diameters.

Figure 2c shows the surface of the photonic thin films after the particle removal and Figure 2d is representative of the internal pore structure.

Two functional monomers commonly used in MIP fabrication, AA and MAA, were tested to investigate the effect of their chemistry on the MIP characteristics. The water contact angle for the polymers was 35 ± 2° and 59 ± 4° for AA and MAA films, respectively. MAA is less hydrophilic due to the methyl moiety than AA, but exhibits better mechanical properties, generally resulting in stronger films, more resistant during handling.

The materials were characterized with respect to their ability to absorb water and swell. Higher water affinity is expected to lead to faster response, as transport of the target is facilitated while a higher degree of swelling translates into a larger variation in the photonic crystal structure, which, in turn, is associated with a more significant optical response.

**Figure 3:**
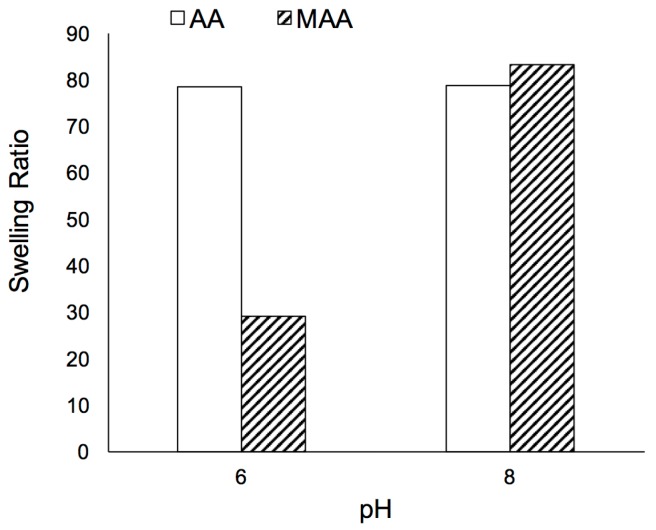
Effect of pH on Swelling Ratio for polyacrylic acid films (clear bars) and polymethacrylic acid films (shaded bars).

AA films presented high swelling ratios both at acidic and basic pH (Figure 3). SR values were lower at pH 6 than at pH 8, most noticeable for MAA. Both polymers are expected to be present in their ionic form, with p*K*_a_ of 4.26 and 4.65 for AA and MAA, respectively [26,27]. However, at the higher pH, the carboxyl group is deprotonated to a higher extent, leading to electrostatic repulsion that causes the polymer to swell more. The contribution of non-polar groups (methyl in MAA) to the overall hydrophobicity of the polymer was outweighed at higher pH by the charge developed as a result of ample deprotonation of the carboxylic groups. The higher SR observed for AA is consistent with the more hydrophilic character of the polymer.

**Figure 4:**
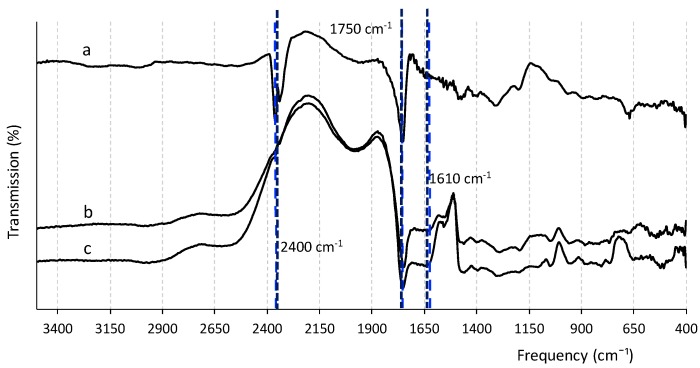
FTIR spectra of the films at different stages of the synthesis process: (**a**) MIP after testosterone removal; (**b**) NIP; (**c**) MIP before testosterone removal.

The chemical functional groups and their interactions with the testosterone molecule after polymerization were investigated by FTIR. The films before and after testosterone removal and the non-imprinted films (NIPs), i.e., same material and morphology but no target molecule added, showed similar IR spectra, but with differences in intensity of some of the bands (Figure 4). MIPs after removal of testosterone had higher intensity of bands compared to those before target removal and NIPs, shown as a control sample. All the imprinted cavities in the MIP before template removal were occupied by testosterone molecules, which lowered the absorbance of functional groups involved in the non-covalent binding; when testosterone was washed from the polymer, those groups were free to vibrate and therefore showed higher adsorption intensity [28]. The spectrum contains various bands: the peak at around 2400 corresponds to the OH group of the carboxylic acid, which is very clearly observed in the MIP samples after target removal. The disappearance of this band in MIP + testosterone samples and in NIPs indicates the formation of the interaction between the carboxylic group of the polymer and testosterone during binding. In addition, the peak at 1610 cm^−1^ may be attributed to the carbonyl group (C=O) of testosterone, as it was not present in the washed MIPs nor in the NIPs’ spectra [29].

RC and IE were calculated from the equilibrium incubation experiments and summarized in Table 1. The AA films presented consistently higher RC and IE than MAA films and therefore were selected as the material to be used in subsequent experiments.

**Figure 5:**
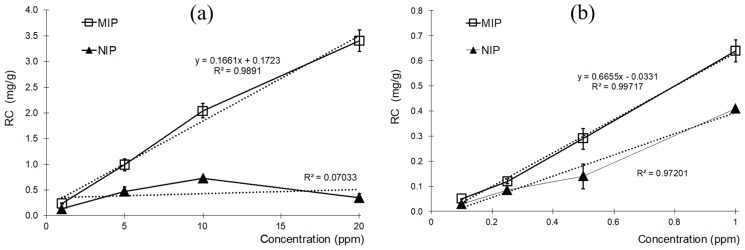
Recognition Capacity for AA-MIP and AA-NIP at higher (**a**) and lower (**b**) range concentrations.

AA MIPs were further tested in an extended concentration range (Figure 5). The MIPs exhibited higher affinity for the template than the NIPs at all testosterone concentrations tested. The RCs increased linearly with concentration, both at the higher, between 1 ppm to 20 ppm, and lower range, 0.1 ppm to 1 ppm.

Testosterone adsorption on the NIP films was lower than for MIPs in all cases, but significant relative to the amount of MIP rebinding for incubations at 1 ppm and below, resulting in poor IE values. The IE reached 10 for the highest concentration of 20 ppm, and declined to 1.6 at the lowest one tested, 0.1 ppm. The average value for IE of AA-MIPs was 3.16.

**Figure 6:**
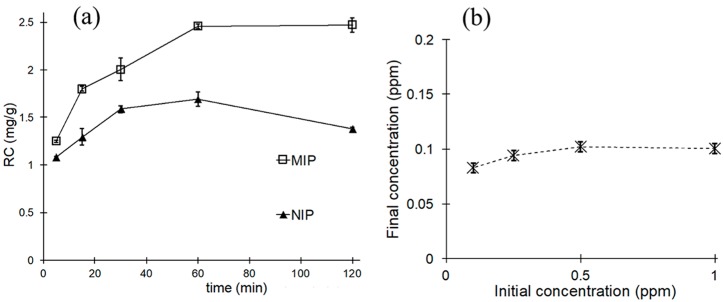
Kinetics of rebinding to unsupported MIP and NIP films, (**a**) initial testosterone concentration 5 ppm; (**b**) desorption of testosterone from MIP films initially incubated at variable concentrations, 0.1 ppm to 1 ppm testosterone.

The kinetics of the testosterone attachment were investigated in order to determine the minimum time required for the MIP to reach equilibrium with the incubation solution. Negligible concentration changes were determined after 60 min of incubation for MIPs (Figure 6a). The stability of the bound testosterone was tested by desorption experiments, where the previously equilibrated films were exposed to pure water. The results showed that the attachment was stable, and minimal transfer of testosterone to the liquid phase was observed (Figure 6b). The lowest amount of testosterone detected after incubation in pure water was 0.083 ppm. This may be due to desorption of previously nonspecific adsorbed testosterone, as this form of interaction is generally weaker than the rebinding into imprinted sites and therefore more likely to reverse when solvent conditions change. The concentrations of testosterone in the final incubation solutions were fairly insensitive to initial solution concentration and quickly reached a plateau at 0.1 ppm. The films were left to dry at room temperature before the second incubation, but a contribution by liquid trapped inside the porous structure cannot be completely ruled out.

### 3.2. Reflectance Analysis

**Figure 7:**
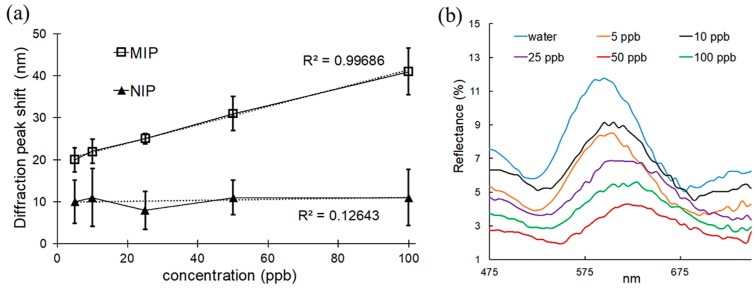
Optical properties supported MIPs: (**a**) diffraction peak shift for MIPs and NIPs after incubation at various concentration of testosterone; (**b**) reflectance spectra of supported MIPs after incubation at various concentration of testosterone.

A simple and inexpensive method for detection of the testosterone stems from the photonic properties of the MIP [30,31,32,33,34]. The periodicity of the porous film resulted in interference in reflection of the incident light. For each film, the wavelength of reflection was correlated to the amount of rebinding of the target; with increasing concentration of testosterone in the incubation solution, the peak wavelength of the reflected light was further shifted to longer wavelengths. As the imprinted nanocavities became increasingly occupied by the target, the internal pore structure of the inverse opal was altered, producing a different optical response (Figure 7). A clear difference could be observed between the behavior of MIPs and NIPs. When MIPs were exposed to increasing concentration of testosterone, the peak of the reflected light gradually shifted to longer wavelengths. However, there was almost no peak displacement for the NIP under the same conditions. We were able to detect low concentrations of testosterone by this technique, from 5 ppb–100 ppb (Figure 7), which is within the normal range of hormone level in adult males. The detection limit was determined to be 4.2 ppb (S/N = 3).

**Figure 8:**
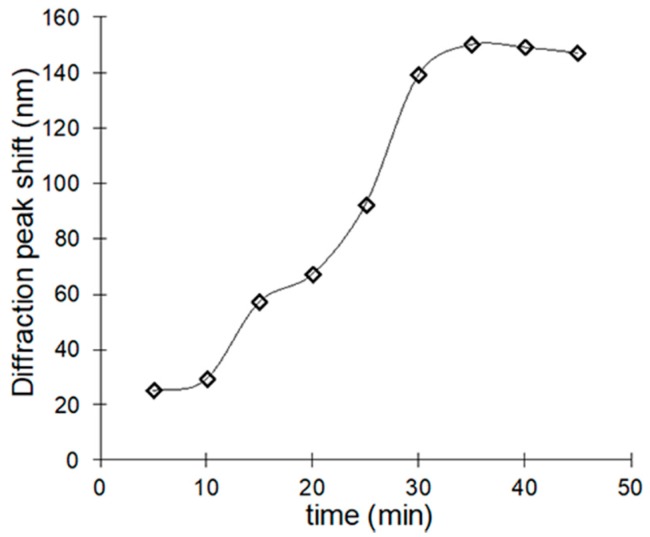
Kinetic response of the testosterone MIP.

**Figure 9:**
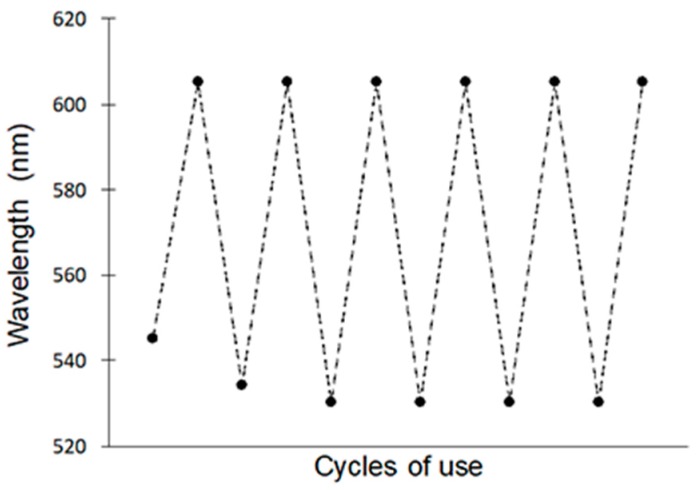
Recoverability of photonic sensor for six cycles of use and regeneration.

The kinetics experiment of the optical response due to the rebinding showed that the peak wavelength became constant for times longer than 30-min incubation in the test solution (Figure 8). The stability of the material was evidenced by the reversibility of the wavelength shifts observed after six cycles of use and regeneration (Figure 9). The clean MIP was promptly recovered after incubating in a 1 ppm solution by eluting the testosterone with an ethanol/acetic acid solution in a ratio 9:1. The highly open internal pore structure of the film facilitated the back diffusion of the testosterone molecules during washing, and a remarkable degree of regeneration of the initial conditions of the film was achieved [15].

Chemicals other than testosterone can potentially bind into the imprinted cavities, resulting in errors due to interferences. To investigate the competitive recognition ability of the MIPs, the response to testosterone was compared to that of a group of selected compounds: estradiol, flutamide, and bisphenol A. Estradiol was selected due to its similar structure to testosterone, while the other two are known to be androgenic endocrine disrupting chemicals, i.e., they interfere with natural receptors of testosterone. The MIPs were tested with different concentrations between 5 ppb and 100 ppb of these chemicals. MIPs were dipped into each solution for 30 min, and spectra was recorded to determine the peak wavelength shift.

**Figure 10:**
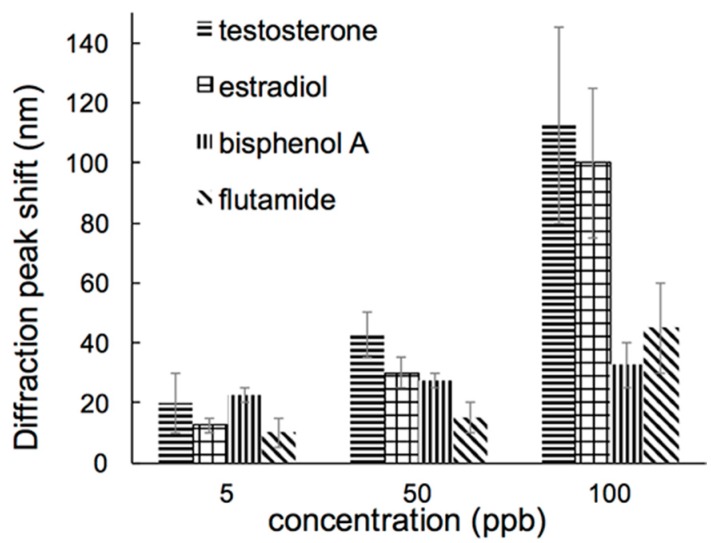
Optical response of the MIPs to testosterone and selected compounds.

The MIPs exhibited different recognition capacity for testosterone and other substances tested (Figure 10). Because of the structural similarities, estradiol induced a response from the MIPs close to that of testosterone. However, selectivity towards testosterone was significantly better when the other two compounds were tested.

## 4. Discussion

Inverse opal imprinted polymers were fabricated from monodisperse silica particle crystals. The thin films were supported on PMMA slides for easy handling as the MIPs can be used as sensors to determine concentration of testosterone, the target molecule, in unknown liquid samples. Although polyMAA resulted in stronger films, polyAA films proved to be a better choice for the intended application, made evident by the significantly higher imprinting efficiency, hydrophilicity and superior water adsorption characteristics. Mechanical strength was not critical as the films were supported by an external material.

The recognition capacity varied linearly with concentration in a wide range of testosterone levels, although using different linear calibration curves based on concentration range is recommended. Some degree of non-specific adsorption was observed for NIPs, in all cases in a lower amount than in MIPs. Testosterone adsorption reached equilibrium after 60 min of incubation with test solutions. However, the optical response of the film presented improved characteristics in relation with its function as testosterone sensors. The diffraction peak wavelength shifted when the MIPs were incubated in testosterone solutions; the shift was measurable after exposures to concentrations down to 5 ppb, reaching the reference levels of testosterone in blood for adult males. The NIPs showed no significant wavelength shift, which implied that the methodology is unaffected by non-specific adsorption. Moreover, the peak wavelength becomes stable after 30-min incubation time, an important improvement over the previous determination. Other hormones with similar chemical structure have the ability to interfere in the accurate determination of testosterone with the MIPs, although the effect can be minimized if the context of the sample is known, i.e., expected estradiol vs. testosterone concentration. Our test experiments represented a worst case scenario, when the response of the interference is compared at the same concentration and in the absence of testosterone. For samples with unknown reference values of hormone concentration, a combination of MIPs targeting each one of the analogs (estradiol, estrogen, testosterone) may provide additional information and help differentiate targets from interfering compounds.

## Figures and Tables

**Table 1:** Recognition capacity and imprinting efficiency of AA and MAA MIPs.

	Concentration (ppm)	RC ^1^ (mg/g)	IE
MAA	1	0.21	1.23
	5	0.51	1.1
	10	0.9	1.5
AA	1	0.22	1.71
	5	0.98	2.13
	10	2	2.77

^1^ average value of at least two samples.
